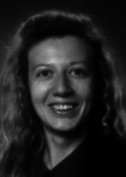# Similar but Still Different: Which Amino Acid Residues Are Responsible for Varying Activities in Type‐III Copper Enzymes?

**DOI:** 10.1002/cbic.202000647

**Published:** 2020-12-11

**Authors:** Ioannis Kampatsikas, Annette Rompel

**Affiliations:** ^1^ Universität Wien Fakultät für Chemie Institut für Biophysikalische Chemie Althanstraße 14 1090 Wien Austria

**Keywords:** activity controllers, gatekeeper residue, oxidases, phenolase activity, tyrosinases, waterkeeper residue

## Abstract

Type‐III copper enzymes like polyphenol oxidases (PPOs) are ubiquitous among organisms and play a significant role in the formation of pigments. PPOs comprise different enzyme groups, including tyrosinases (TYRs) and catechol oxidases (COs). TYRs catalyze the *o*‐hydroxylation of monophenols and the oxidation of *o*‐diphenols to the corresponding *o*‐quinones (EC 1.14.18.1). In contrast, COs only catalyze the oxidation of *o*‐diphenols to the corresponding *o*‐quinones (EC 1.10.3.1). To date (August 2020), 102 PDB entries encompassing 18 different proteins from 16 organisms and several mutants have been reported, identifying key residues for tyrosinase activity. The structural similarity between TYRs and COs, especially within and around the active center, complicates the elucidation of their modes of action on a structural basis. However, mutagenesis studies illuminate residues that influence the two activities and show that crystallography on its own cannot elucidate the enzymatic activity mode. Several amino acid residues around the dicopper active center have been proposed to play an essential role in the two different activities. Herein, we critically review the role of all residues identified so far that putatively affect the two activities of PPOs.

## Graphical abstract

**Cause and effect**: The mode of action of PPOs is still elusive. Here, we summarize the current understanding of the amino acids located around the dicopper active center and the mutations that were applied to these residues that control the substrate selectivity and activity of PPOs, and thereby decide whether a PPO reacts as a tyrosinase or a catechol oxidase.

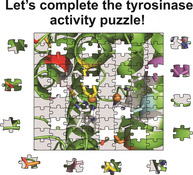

## 1 Type‐III Copper Proteins

### 1.1 Functionality of type‐III copper proteins

**Figure 1:**
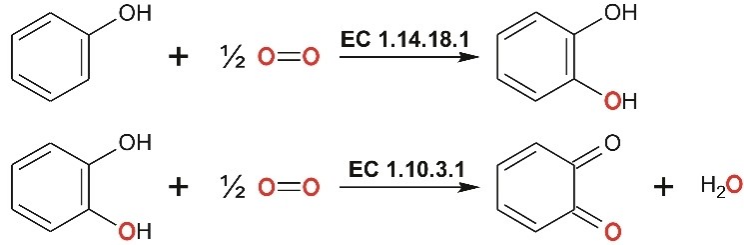
Reactions catalyzed by PPOs. Top: *o*‐hydroxylation of monophenols to *o*‐diphenols (monophenolase activity, EC 1.14.18.1); bottom: oxidation of *o*‐diphenols to the corresponding *o*‐quinones (diphenolase activity, EC 1.10.3.1). TYRs catalyze both reactions, whereas COs catalyze only the diphenolase reaction.

Polyphenol oxidases (PPOs) and hemocyanins (HCs) constitute the type‐III copper family,[^1^, ^2^, ^3^] and are involved in oxygen activation, and oxygen transport, respectively. PPOs can be subdivided into tyrosinases (TYRs), catechol oxidases (COs), and aurone synthase (AUS), whereby each of these three enzyme types contains a crystallographically verified type‐III copper center. TYRs catalyze the *o*‐hydroxylation of monophenols and the subsequent oxidation of *o*‐diphenols to the corresponding *o*‐quinones (monophenolase or cresolase activity; EC 1.14.18.1, Figure 1, top). In contrast, COs can only catalyze the oxidation of *o*‐diphenols (diphenolase or catecholase activity: EC 1.10.3.1, Figure 1, bottom) as they do not react with monophenols.[^4^, ^5^] AUS is an enzyme that is functionally in‐between TYRs and COs, and catalyzes the formation of aurones *in vivo*. However, wild‐type AUS from *Coreopsis grandiflora* (*Cg*AUS_wt_) exhibits a weak monophenolase activity toward its natural substrate isoliquiritigenin,^[6]^ whereas it does not react with the classical TYR substrates l‐tyrosine and tyramine, and is therefore classified as a CO.[^6^, ^7^]

HCs are oxygen‐transport proteins in the hemolymph of arthropods and mollusks,^[8]^ and usually lack hydroxylase or oxidase activity on phenolic compounds. Detergents like sodium dodecyl sulfate (SDS), however, trigger monophenolase and diphenolase activity in HCs[^9^, ^10^] and pro‐PPOs,[^11^, ^12^, ^13^] and thereby impart tyrosinase activity to these enzymes (Figure 1). SDS is assumed to change the conformation of type‐III copper proteins like HCs^[10]^ and PPOs,^[14]^ thereby enabling substrates to enter the active center. SDS has been used widely in enzymatic activation of pro‐PPOs.[^13^, ^15^, ^16^] However, the lack of structural information on the mode of interaction between SDS and pro‐PPOs prevents clarification of the specific conformation changes upon activation. The only available structural data (PDB ID: 4D87) were collected for the active TYR from *Bacillus megaterium* (*Bm*TYR) in the presence of SDS, where SDS was detected 13.4 Å apart from the dicopper active center and in proximity (3.0 Å) to residue R209 (second activity controller, see Section 2.5).^[14]^ In the same study, SDS increased the monophenolase and diphenolase activity compared to the enzyme‘s natural activity, and therewith significantly influenced the substrate specificity of *Bm*TYR.

### 1.2 Polyphenol oxidases (PPOs)

#### 1.2.1 Occurrence of PPOs

PPOs (TYRs and COs)^[17]^ are widely distributed in nature and are found in bacteria,[^18^, ^19^] archaea,^[20]^ fungi,[^21^, ^22^] plants,[^17^, ^23^, ^24^] and mammals.[^25^, ^26^, ^27^] PPOs are essential for the biosynthesis of melanin and consequently responsible for the enzymatic browning of most fruits and vegetables,[^23^, ^24^] and the pigmentation in mammals.[^26^, ^27^, ^28^] In insects, PPOs are involved in the sclerotization of the exoskeleton.^[29]^ PPOs also play a significant role in various diseases such as albinism,^[30]^ melanoma,^[31]^ and Parkinson.^[32]^

#### 1.2.2 Domains of PPOs

Plant PPOs are expressed as pre‐pro‐enzymes *in vivo* as they contain a signal peptide (pre‐) and a C‐terminal domain (pro‐). Specifically, they are expressed as ∼64–68 kDa proteins consisting of three domains: a chloroplastic transit peptide containing a thylakoid signal peptide (∼4‐9 kDa, present in the majority of plant PPOs),^[21]^ a catalytically active domain (∼37–42 kDa), and a C‐terminal domain (∼15–19 kDa active site‐shielding domain).^[23]^ In contrast, most of the bacterial PPOs do not contain a signal peptide and the majority of them lack a C‐terminal domain.[^18^, ^21^] However, some bacterial PPOs such as *Burkholderia thailandensis* (*Bt*TYR), contain a C‐terminal domain similar to that in plant PPOs.^[33]^ Fungal PPOs, like bacterial PPOs, mostly lack a signal peptide, but the majority of them contain a C‐terminal domain.[^12^, ^21^, ^22^] The sequence of insect prophenoloxidases does not start with a signal peptide but with an N‐terminal domain.[^25^, ^29^] Mammalian PPOs harbor an N‐terminal signal peptide and their C‐terminal domain is involved in membrane integration.^[26]^

#### 1.2.3 *The dicopper center in the catalytically active domain in PPOs*

**Figure 2:**
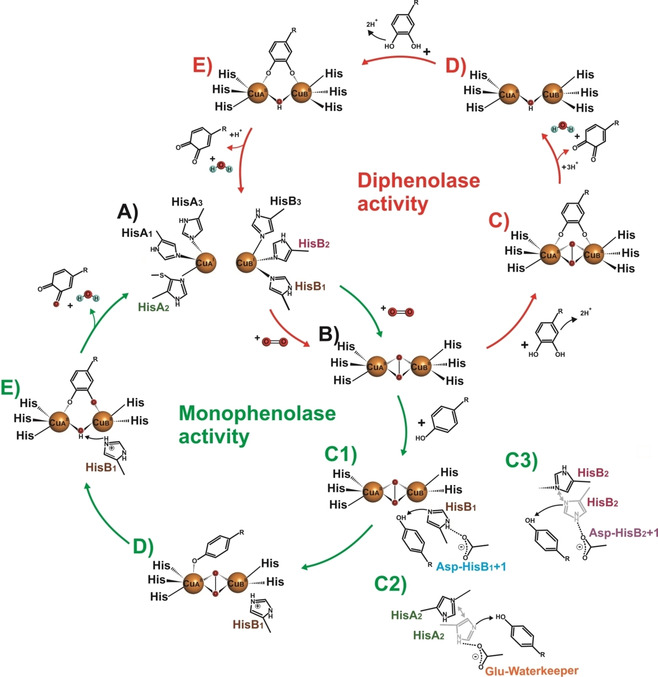
Schemes of the mono‐(green cycle) and diphenolase (red cycle) activity of PPOs. Α) The deoxy‐form of the type‐III copper center (Cu^I^‐Cu^I^) is the starting point for both activities. It binds molecular oxygen and thereby switches to the catalytically active oxy*‐*form (Cu^II^‐Cu^II^). Β) Monophenolase activity (green): C1), C2), C3) residues located within or around the dicopper center (HisB_1_+1, HisB_2_+1, and the waterkeeper residue) enhance the basicity of the conserved copper‐coordinating histidines (HisB_1_, HisB_2_, and HisA_2_), which then deprotonate the incoming monophenolic substrates. D) The deprotonated monophenol, ready for the catalytic reaction, interacts with the oxy‐form of the type‐III copper center. E) *ortho*‐hydroxylation of the phenolate by an electrophilic aromatic substitution and the subsequent two‐electron oxidation of the diphenolic intermediate yield the final *ortho*‐quinone product, and one molecule of water. During the two‐electron oxidation step, the PPO copper center is reduced to its deoxy‐form, closing the catalytic monophenolase cycle.^[45]^ Diphenolase activity (red): C) The diphenolic substrate is oxidized to the corresponding quinone by the dicopper center, which transitions from the oxy‐ to the met‐form. D) The met‐form accepts diphenolic substrates and converts them to the corresponding quinones. E) Similarly to the monophenolase activity, the PPO copper center is reduced to its deoxy‐form during substrate oxidation, thereby closing the catalytic diphenolase cycle.^[2]^

In all PPOs, each copper ion is coordinated by three imidazole nitrogens of conserved histidines (Figure 2A). The type‐III center can exist in its deoxy‐ (Cu^I^ Figure 2A), oxy‐ (Cu^II^, Figure 2B), or met‐form (Cu^II^, Figure 2E), respectively. In the deoxy‐form, the distance between the two copper atoms is about 4.1–4.6 Å, whereas in the met‐ and oxy‐form, the Cu−Cu distances are about 3.2–4.0 Å and 2.8–3.2 Å, respectively.^[34]^ Mushroom TYR, purified source, exists as a mixture of oxy‐ (15 %) and met‐form (85 %),^[35]^ and PPOs are predominantly characterized in their resting met‐form in which a hydroxide or water molecule bridges the two copper ions in their cupric state (Cu^II^).[^1^, ^2^] The deoxy‐form, in which the copper ions are in the cuprous state (Cu^I^), can bind molecular dioxygen in a side‐on bridging geometry (μ‐η^2^ : η^2^), yielding the oxy‐form, with the copper ions being in the oxidation state +2 (Cu^II^). Upon saturation with oxygen, the oxy‐form of the type‐III copper center exhibits two characteristic ligand‐to‐metal charge‐transfer bands in the UV/Vis spectrum, one at ∼345 nm and a second one at ∼580 nm, which is ∼20 times less intense.[^1^, ^36^, ^37^, ^38^, ^39^] The oxy‐form (Figure 2B) can bind and convert mono‐ as well as diphenols, while the met‐form (Figure 2E) of PPOs only converts diphenols to the corresponding *o*‐quinones by a two‐electron oxidation reaction (Figure 2).[^40^, ^41^, ^42^] After the conversion of mono‐ or diphenols to the corresponding *o*‐quinones, PPOs are recycled back to their deoxy‐form (Figure 2A) and the enzymes are ready to start a new catalytic cycle (Figure 2).[^21^, ^43^]

## 2 What Causes the Mono‐ *versus* Diphenolase Activity in Type‐III Copper Proteins?

**Figure 3:**
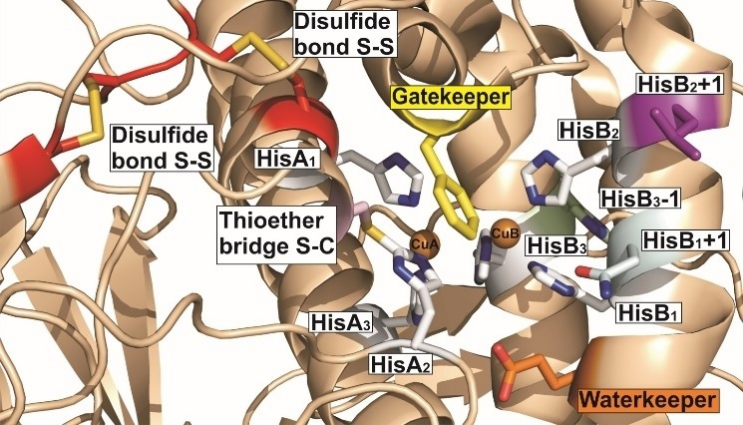
Catalytic residues within and around the active center of PPOs. The crystal structure of TYR from *Juglans regia* (*Jr*PPO1, PDB ID: 5CE9) is used as a representative example.[^51^, ^52^] The six conserved histidines HisA_1_, HisA_2_, and HisA_3_ of CuA and HisB_1_, HisB_2_, and HisB_3_ of CuB are depicted as sticks (carbon: white and nitrogen: blue). The seventh His, HisB_3_‐1 (light green), waterkeeper residue (orange), gatekeeper residue (yellow), and the two activity controllers, HisB_1_+1 (gray) and HisB_2_+1 (purple) are highlighted. The thioether bridge (pink) connects the second conserved histidine of CuA (HisA_2_) to an adjacent cysteine residue, whereas the two disulfide bonds are presented in red.

For decades, researchers are searching for the structural differences between TYRs and COs that can explain their different catalytic activities.[^34^, ^44^] The high structural similarity of the active site among PPOs has been documented by 102 PDB entries covering 18 different PPOs from 16 organisms (as of August 2020). Despite, the relatively high number of PPO structures, it is hitherto impossible to distinguish between TYRs and COs, and to predict their activities solely on a structural basis, which reduces the interest in crystallizing new type‐III copper enzymes to address this important question. Our current understanding is that the hydroxylation of monophenols by PPOs depends on two prerequisites: i) the dicopper center has to be in its oxy‐form (Figure 2B) and ii) the monophenolic substrate must be deprotonated before binding to the dicopper center (Figure 2C1–C3).[^34^, ^45^, ^46^] Substrate deprotonation is supposed to be controlled *via* a conserved water molecule. However, mutation studies targeting amino acids within and close to the active site (Figure 3) have shown that a number of amino acid residues influence the two different activities (hydroxylation and oxidation), which gives an entirely new perspective to the deprotonation of monophenolic compounds by the conserved His of the dicopper center as detailed in Section 2.

### 2.1 The conserved histidines

#### 2.1.1 *Classical proposed roles of the conserved histidines*

The six histidines (HisA_1_, HisA_2_ HisA_3_, HisB_1_, HisB_2_, and HisB_3_), coordinate the two copper ions and are conserved among all type‐III copper proteins. Crystallographic investigations showed that the two copper ions are flexible and can thus assume different positions within the active center. Due to this flexibility the conserved histidines can temporarily lose their interactions with the copper ions. This was impressively demonstrated in the crystal structure of *Streptomyces castaneoglobisporus* TYR (*Sc*TYR) in which CuA and CuB were found at three and two different positions, respectively.^[47]^ Similarly, in *Aspergillus oryzae* TYR (*Ao*TYR), the two copper ions were crystallographically detected at different positions.^[48]^ According to the authors of both studies, the migration of the two copper ions is associated with the binding of the incoming substrate and represents an essential feature for the hydroxylation of monophenolic substrates. CuB in *Agaricus bisporus* PPO4 (*Ab*PPO4 PDB ID: 4OUA) also shows an alternative conformation, which is stabilized by the seventh His residue (HisB_3_‐1, Figure 3). The seventh His residue, which is conserved in plant, fungal, and bacterial PPOs, is involved in CuB binding in *Ab*PPO4. Therefore, CuB interacts with four histidines, and the seventh His can also be involved in CuB binding.[^49^, ^50^]

#### 2.1.2 Mutations of the conserved histidines

In *Cg*AUS the CuA‐ (His93, His116, and His125) and CuB‐coordinating (His252, His256, and His286) histidines (Figure 3) were mutated to Ala. Out of the six mutants only His93Ala and His256Ala still had diphenolase activity, while the rest of the mutants lacked enzymatic activity. Furthermore, the mutants (His93Ala, His116Ala, and His125Ala) contained only one copper ion per enzyme molecule (Table 1).^[53]^ In a study on human tyrosinase (*Hs*TYR) the three conserved histidines of CuB were also mutated to Ala (His363Ala, His367Ala, and His390Ala).^[54]^ As a result, the mutants His363Ala and His367Ala lost their tyrosinase activity completely, probably due to the loss of the copper ions. In contrast, the His390Ala mutant showed enhanced copper‐binding as its copper content was approximately 2.5 times higher than that of the wild‐type enzyme (Table 1).^[54]^ In a second mutation study on *Hs*TYR all of the six conserved histidines (His180, His202, His211, His363, His367, and His390) of *Hs*TYR were replaced one by one with alanine and all the mutations impaired their monophenolase (l‐tyrosine) and diphenolase (l‐DOPA) activity (Table 1).^[55]^ Single mutations of the six conserved histidines (His63, His84, His93, His290, His294, and His333) to Asn in *Ao*TYR (melO) led to the loss of monophenolase (l‐tyrosine) as well as diphenolase (l‐DOPA) activity, whereby all mutants contained only about one copper ion per enzyme (Table 1).^[56]^ In another study using the bacterial TYR from *Streptomyces glaucescens* (*Sg*TYR) the six conserved histidines (His37, His53, His62, His189, His193, and His215) were mutated to Gln (His37, His53, His193, and His215) and Asn (His62 and His189).^[57]^ The diphenolase activity on l‐DOPA of the mutants His193Gln and His215Gln was 6250‐ and 2778‐fold lower than that of the wild type, whereas the remaining mutants His37Gln, His53Gln, His62Asn, and His189Asn were almost inactive on l‐DOPA (Table 1).^[57]^ Furthermore, the monophenolase activity of *Sc*TYR on l‐tyrosine was abolished by mutating its conserved histidine His63 (HisA_3_) to Phe (Table 1).^[47]^ According to these mutagenesis studies, the conserved histidines are structurally and chemically essential residues in PPOs. The copper content of the mutants was reduced and therefore, the majority of the mutants affecting the six conserved histidines resulted in either a partially active or completely inactive enzyme.

**Table 1:** Summary of the mutations to the six conserved histidines: effects on PPOs′ copper content and diphenolase/monophenolase activity. The modification of copper content and the activities′ rate are described compared to the wild type of the corresponding enzymes.

Conserved histidines					
Enzyme	Mutant	Diphenolase activity	Monophenolase activity	Copper %	Ref.
*Cg*AUS	His93Ala (HisA_1_)	−178‐fold (butein: 62 μmol/(l×min) −240‐fold (fisetin: 92 μmol/(l×min) −575‐fold (4‐*tert*‐butylcatechol: 218 μmol/(l×min)	n.i.	50	^[53]^
	His116Ala‐(HisA_2_)	n.d. (butein, fisetin, and 4‐*tert*‐butylcatechol)	n.i.	50	
	His125Ala‐(HisA_3_)	n.d. (butein, fisetin, and 4‐*tert*‐butylcatechol)	n.i.	50	
	His252Ala‐(HisB_1_)	n.d. (butein, fisetin, and 4‐*tert*‐butylcatechol)	n.i.	n.i.	
	His256Ala‐(HisB_2_)	−409‐fold (butein: 27 μmol/(l×min) n.d. (fisetin and 4‐*tert*‐butylcatechol)	n.i.	n.i.	
	His286Ala‐(HisB_3_)	n.d. (butein, fisetin, and 4‐*tert*‐butylcatechol)	n.i.	n.i.	
*Hs*TYR	His363Ala‐(HisB_1_)	n.i.	n.d. (l‐tyrosine)	0	^[54]^
	His367Ala‐(HisB_2_)	n.i.	n.d. (l‐tyrosine)	50	
	His390Ala‐(HisB_3_)	n.i.	n.d. (l‐tyrosine)	250	
	His180Ala‐(HisA_1_)	−1.20‐fold (l‐DOPA: 28.5 s^−1^)	−1.79‐fold (l‐tyrosine: 0.34 s^−1^)	n.i.	^[55]^
	His202Ala‐(HisA_2_)	−1.14‐fold (l‐DOPA: 30.0 s^−1^)	−1.45‐fold (l‐tyrosine: 0.42 s^−1^)	n.i.	
	His211Ala‐(HisA_3_)	−1.05‐fold (l‐DOPA: 32.4 s^−1^)	−1.22‐fold (l‐tyrosine: 0.50 s^−1^)	n.i.	
	His363Ala‐(HisB_1_)	−1.98‐fold (l‐DOPA: 17.2 s^−1^)	−1.38‐fold (l‐tyrosine: 0.44 s^−1^)	n.i.	
	His367Ala‐(HisB_2_)	−2.16‐fold (l‐DOPA: 15.8 s^−1^)	−1.27‐fold (l‐tyrosine: 0.48 s^−1^)	n.i.	
	His390Ala‐(HisB_3_)	−1.15‐fold (l‐DOPA: 29.7 s^−1^)	−1.17‐fold (l‐tyrosine: 0.52 s^−1^)	n.i.	
*Ao*TYR^[a]^	His63Asn‐(HisA_1_)	n.d. (l‐DOPA)	n.d. (l‐tyrosine)	45	^[56]^
	His84Asn‐(HisA_2_)	n.d. (l‐DOPA)	n.d. (l‐tyrosine)	35	
	His93Asn‐(HisA_3_)	n.d. (l‐DOPA)	n.d. (l‐tyrosine)	50	
	His290Asn‐(HisB_1_)	n.d. (l‐DOPA)	n.d. (l‐tyrosine)	30	
	His294Asn‐(HisB_2_)	n.d. (l‐DOPA)	n.d. (l‐tyrosine)	40	
	His333Asn‐(HisB_3_)	n.d. (l‐DOPA)	n.d. (l‐tyrosine)	40	
*Sg*TYR	His37Gln‐(HisA_1_)	−12500‐fold (l‐DOPA: ∼0.2 units/mg)	n.i.	55	^[57]^
	His53Gln‐(HisA_2_)	−12500‐fold (l‐DOPA: ∼0.2 units/mg)	n.i.	45	
	His62Asn‐(HisA_3_)	−12500‐fold (l‐DOPA: ∼0.2 units/mg)	n.i.	55	
	His189Asn‐(HisB_1_)	−12500‐fold (l‐DOPA: ∼0.2 units/mg)	n.i.	90	
	His193Gln‐(HisB_2_)	−6250‐fold (l‐DOPA: 0.4 units/mg)	n.i.	50	
	His215Gln‐(HisB_3_)	−2778‐fold (l‐DOPA: 0.9 units/mg)	n.i.	85	
*Sc*TYR	His63Phe‐(HisA_3_)	n.i.	n.d. (l‐tyrosine)	n.i.	^[47]^

[a] Refers to *Ao*TYR (mel0‐Q00234). n.d.: no detected activity, n.i.: no information from the particular study, entries in μmol/(l×min) refer to volumetric activity, s^‐1^ to k_cat_ values, and units/mg to specific enzymatic activity. One unit of enzymatic activity is defined as that amount of enzyme that catalyzes the formation of one μmol of product (*ortho*‐quinone) per minute under reaction conditions optimized for quickest conversion of the respective educt.

### 2.2 The gatekeeper residue

#### 2.2.1 *Classical proposed roles of* the gatekeeper residue

The so‐called gatekeeper residue (Figure 3) is located above CuA and was first identified and described in *Ipomoea batatas* CO (*Ib*CO), in which it is a Phe (Phe261).^[58]^ Phe as the gatekeeper residue is conserved among plant PPOs (i. e., *Vitis vinifera* PPO (*Vv*PPO),^[59]^ *Jr*TYR,[^52^, ^60^] *Cg*AUS,[^6^, ^7^, ^11^, ^45^] *Malus domestica* PPO1 (*Md*PPO1),[^15^, ^61^, ^62^] and *Solanum lycopersicum* PPO (*Sl*PPO1)^[63]^). PPOs from fungi,[^12^, ^50^] bacteria^[18]^ or animals[^64^, ^65^] have different amino acid residues at the gatekeeper position, such as valine,[^19^, ^66^, ^67^] glycine,^[68]^ alanine,[^12^, ^49^, ^50^] glutamic acid,^[25]^ asparagine^[33]^ or even threonine as in *Homo sapiens* tyrosinase‐related protein‐1 (*Hs*TYRP1).^[27]^ Because the first crystal structure of a CO (*Ib*CO)^[58]^ contained a bulky Phe at the gatekeeper position, while the first TYR structures (*Bm*TYR^[19]^ and *Sc*TYR^[68]^) contained smaller residues (i. e., valine and glycine), the bulky gatekeeper residue was believed to be responsible for the lack of monophenolase activity in COs. In this regard, it was postulated that a bulky gatekeeper sterically prevents monophenolic substrates from accessing CuA, which was believed to be responsible for monophenolase activity.^[58]^ Another theory stated that in plant PPOs, the gatekeeper residue, together with the thioether bridge (Figure 3), impeded the rotation, and thus the activation of monophenolic substrates. Based on this theory, in bacterial and fungal PPOs, the rotation of the monophenolic substrate would be only possible due to the lack of a bulky Phe at the gatekeeper position.[^12^, ^19^] These theories were later refuted by the crystal structures of different plant TYRs, *Jr*PPO1,[^51^, ^52^] *Md*PPO1,[^15^, ^61^, ^62^] and *Sl*PPO1,^[63]^ unveiling that also TYRs can harbor a bulky gatekeeper residue. Despite having a Phe gatekeeper, *Jr*PPO1 and *Md*PPO1 are highly active on the classic monophenolic substrates l‐tyrosine and tyramine.[^15^, ^51^, ^52^, ^60^, ^61^, ^62^]

#### 2.2.2 Mutations of the gatekeeper residue

The gatekeeper residue (Phe260) of walnut TYR (*Jr*PPO1) was mutated to Gly and the resulting Phe260Gly mutant remained a weak TYR, as it still reacted with monophenolic substrates such as tyramine, but with a 76‐fold reduced activity compared with that of the wild type. However, the activity of the mutant towards the diphenols dopamine and l‐DOPA was also 12 times lower than that of the wild type (Table 2).^[69]^

**Table 2:** Summary of the mutations to the gatekeeper residue: effects on PPOs′ copper content and diphenolase/monophenolase activity. The modification of copper content and the activities′ rate are described compared to the wild type of the corresponding enzymes.

Gatekeeper residue					
Enzyme	Mutant	Diphenolase activity	Monophenolase activity	Copper %	Ref.
*Jr*PPO1	Phe260Gly	−12‐fold (l‐DOPA: 9.1 s^−1^) −12‐fold (dopamine: 7.4 s^−1^)	−76‐fold (tyramine: 0.3 s^−1^)	50	^[69]^
*To*PPO2	Phe260Leu	−5.0‐fold (4‐methylcatechol) n.d.(dopamine)	−4.0‐fold (*p‐*cresol and tyramine)	n.i.	^[70]^
	Phe260Pro	n.d. (4‐methylcatechol and dopamine)	n.d. (*p‐*cresol and tyramine)	n.i.	
	Phe260Gly	n.d. (4‐methylcatechol and dopamine)	n.d. (*p‐*cresol and tyramine)	n.i.	
*Md*PPO1	Phe259Ala	slower (dopamine: *in crystallo* activity)	n.d. (tyramine: *in crystallo* activity)	n.i.	^[61]^
*Cg*AUS	Phe273Ala	−2760‐fold (butein: 4 μmol/(l×min) n.d. (fisetin and 4‐*tert* butylcatechol)	n.i.	100	^[53]^
	Phe273His	−57‐fold (dopamine: 9.68 s^−1^)	n.d. (tyramine)	31	^[45]^
	Phe273Asp	−750‐fold (dopamine: 0.74 s^−1^)	n.d. (tyramine)	21	
	Phe273Leu	−5.0‐fold (dopamine: 109 s^−1^)	++ (tyramine: 0.27 s^−1^)	89	
	Phe273Ala	−640‐fold (dopamine: 0.87 s^−1^)	n.d. (tyramine)	10	
*Bm*TYR	Val218Phe	−2.0‐fold (l‐DOPA: 21.0 s^−1^)	+4.0‐fold (l‐tyrosine: 16.7 s^−1^)	100	^[71]^
	Val218Gly	+2.0‐fold (l‐DOPA: 73.3 s^−1^)	+8.0‐fold (l‐tyrosine: 31.1 s^−1^)	100	

n.d.: no detected activity, n.i.: no information from the particular study, ++: generation of monophenolase activity that was not present in the wild type, values in μmol/(l×min) refer to volumetric activity while s^‐1^ indicates a k_cat_ value.

The gatekeeper residue Phe260 of a TYR from *Tarraxacum officinale* (*To*PPO2) was mutated to Leu, Gly, and Pro. Of these mutants, only Phe260Leu was active, while Phe260Gly and Phe260Pro showed no activity on the substrates examined (i. e., *p*‐cresol, tyramine, 4‐methylcatechol, and dopamine). Kinetic data of the Phe260Leu mutant indicated that the monophenolase activity on *p*‐cresol and tyramine decreased about fourfold, whereas the diphenolase activity on 4‐methylcatechol was reduced approximately fivefold compared with that of the wild type (Table 2).^[70]^

In addition, the gatekeeper residue (Phe259) of apple TYR *Md*PPO1 was mutated to Ala (Phe259Ala), and the mutant was crystallized. Crystals of the wild type and Phe259Ala mutant were incubated in crystallization drops containing the substrates tyramine or dopamine and SDS to activate the pro‐form of the enzymes. The crystals of the wild‐type enzyme reacted with both the monophenolic tyramine and the diphenolic dopamine, as evidenced by a color change of the protein crystal from colorless to brownish. In contrast, Phe259Ala was only slightly active on dopamine and inactive on tyramine (Table 2).^[61]^

In the CO *Cg*AUS, the gatekeeper residue Phe273 was mutated to Ala and the mutation reduced its diphenolase activity with butein 2760‐fold, whereas it was inactive with fisetin and 4‐*tert*‐butylcatechol (Table 2).^[53]^ In a recent study, the gatekeeper Phe273 residue of the same enzyme was mutated to Ala, Leu, Asp, and His. Phe273Ala, Phe273His, and Phe273Asp were inactive on monophenols, although copper ions were present in their active centers^[45]^. In contrast, Phe273Leu showed monophenolase activity against tyramine and l‐tyrosine. The four mutants targeting Phe273 showed reduced specificity for dopamine, thus suggesting that Phe at the gatekeeper position stabilizes the phenolic substrate better than Ala, Leu, or Asp. This stabilization originates from hydrophobic π‐π interactions between the phenol group of the substrate and the phenyl group of the gatekeeper and the imidazole group of HisB_2_.[^7^, ^52^] However, these π‐π interactions cannot be the sole basis for monophenolase activity as the mutant Phe273Leu was active on tyramine and l‐tyrosine (Table 2).^[52]^

The gatekeeper residue Val218 in *Bm*TYR was subjected to two single mutations (Val218Phe and Val218Gly). A reduction in monophenolase activity was expected for the Val218Phe mutant due to the inhibition of substrate entry into the active dicopper center by the bulky Phe at the gatekeeper position. However, the monophenolase activity on l‐tyrosine of both mutants, Val218Phe and Val218Gly increased by about four‐ and eightfold, respectively, compared to that of the wild type. The diphenolase activity on l‐DOPA of Val218Phe decreased about twofold, whereas that of Val218Gly increased about twofold compared to the wild type (Table 2).[^18^, ^71^] Mutations to the gatekeeper residue revealed the significance of the bulky Phe to escort the incoming substrate to the active site through π‐π interactions in plant PPOs. In all the mutants in which the Phe gatekeeper residue was changed to a smaller amino acid, the enzyme‘s original activity was impaired. However, the Phe273Leu mutant of *Cg*AUS produced monophenolase activity with the classical monophenolic substrates (l‐tyrosine and tyramine), indicating that the gatekeeper residue (Phe) does impede the tyrosinase activity.^[45]^ The gatekeeper residue therefore has a dual function and either supports (π‐π interactions with Phe or free entry for the substrate with a small amino acid at this position) or inhibits (blocks the access) the substrate entry in plant PPOs.

### 2.3 Waterkeeper residue

#### 2.3.1 *Classical proposed roles for* the waterkeeper residue

The theory that a conserved glutamic acid (waterkeeper residue) at the entrance of the active site is responsible for the deprotonation of monophenolic substrates was proposed a few years ago.[^25^, ^65^] A recent study claimed that a highly conserved water molecule, which is activated by the waterkeeper (Glu)[^18^, ^72^] deprotonates incoming substrates (Figure 3). The waterkeeper residue Glu is conserved among almost all biochemically characterized PPOs, except for *A. oryzae* CO (*Ao*CO, PDB ID: 4J3P), in which the waterkeeper residue is Gln.^[67]^ The different waterkeeper residues in *Ao*CO (Gln) and *Ao*TYR (Glu) confirm the theory that the different activities of these two *Ao*PPOs are due to a different waterkeeper.[^66^, ^67^] However, *Ao*CO is an exception because other COs like *Ib*CO contain the conserved Glu at the waterkeeper position. Therefore, the waterkeeper Gln seems not to be the sole reason for the weak monophenolase activity in *Ao*CO.

#### 2.3.2 Mutations of the waterkeeper residue

The waterkeeper residue (Glu364) of *Anopheles gambiae* prophenoloxidase (*Ag*proPO) was mutated to Gln and the mutation impaired both monophenolase and diphenolase activity by 14‐fold and 7.7‐fold, respectively, compared with that of the wild type (Table 3).^[65]^

**Table 3:** Summary of the mutations to the waterkeeper residue: effects on PPOs′ copper content and diphenolase/monophenolase activity. The modification of copper content and the activities′ rate are described compared to the wild type of the corresponding enzymes.

Waterkeeper residue					
Enzyme	Mutant	Diphenolase activity	Monophenolase activity	Copper %	Ref.
*Ag*proPO	Glu364Gln	−14‐fold (dopamine)	‐7.7‐fold (tyramine)	n.i.	^[65]^
*To*PPO2	Glu235Asp	−1.5‐fold (4‐methylcatechol) −3.2‐fold (dopamine)	n.i.	n.i.	^[70]^
	Glu235Gln	n.d. (4‐methylcatechol and dopamine)	n.i.	n.i.	
*Cg*AUS	Glu248Ala	−200‐fold (dopamine: 2.79 s^−1^)	n.d. (tyramine)	15	^[45]^
	Glu248Lys	n.d. (dopamine)	n.d. (tyramine)	0	

n.d.: no detected activity, n.i.: no information from the particular study, the entry in s^−1^ refers to the k_cat_ value.

In *To*PPO2 (a TYR), the waterkeeper residue Glu235 was mutated to Asp and Gln. The Glu235Asp mutant exhibited deteriorated diphenolase activity on the diphenolic substrates 4‐methylcatechol and dopamine, while Glu235Gln mutant was completely inactive on these substrates (Table 3).^[70]^

The waterkeeper mutants Glu248Ala and Glu248Lys in *Cg*AUS emphasized the importance of a negatively charged amino acid (Glu) at this position. The diphenolase activity on dopamine of Glu248Ala was 200 times lower than that of the wild type, whereas the Glu248Lys mutant was completely inactive against all substrates tested, probably due to the lack of copper ions (Table 3).^[45]^ Mutagenesis studies targeting the waterkeeper residue of different PPOs proved the importance of Glu at this position for tyrosinase activity. The conversion of Glu to either an uncharged or to a positively charged amino acid eliminates the enzyme's tyrosinase activity. Even the conversion to the shorter Asp in *To*PPO2 has significant negative effects and reduces the PPO's activity (Table 3). Thus, the conserved Glu in PPOs is vital to stabilize water or, in general, a water network around the active center. The single mutations to the waterkeeper are detrimental to the PPOs′ tyrosinase activity, and none of them enhances the activity (Table 3).

### 2.4 The first activity controller (HisB_1_+1)

#### 2.4.1 *Classical proposed roles of* the first activity controller

Various amino acids are located at the position of the first activity controller (HisB_1_+1, Figure 3), which include Ile, Thr, and Gly in the COs *Ib*CO,[^37^, ^58^] *Cg*AUS,[^6^, ^7^, ^11^] and *Ao*CO,^[67]^ respectively, whereas an Asn often, but not exclusively, occupies this position in TYRs. Amino acids other than Asn at HisB_1_+1 were also detected in *Ab*PPO4 (Asp),[^12^, ^22^, ^50^] *Larrea tridentata* PPO (*Lt*PPO, Gly),^[73]^ the two apple TYRs *Md*PPO1 and *Md*PPO3 (Ala and Gly, respectively),[^15^, ^61^] and tomato TYR *Sl*PPO1 (Ser).^[63]^ It was initially speculated that an Asn residue at position HisB_1_+1 is required in TYRs for monophenolase activity because it stabilizes the highly conserved water molecule for activation so that it can act as a base for substrate deprotonation (as described in Section 2.3.1). This theory, however, is being falsified by TYRs that contain another amino acid at this position.^[15]^

#### 2.4.2 Mutations of the first activity controller (HisB_1_+1)

A mutagenesis study targeting the first activity controller Ala243 of apple TYR (*Md*PPO1) showed that both monophenolase and diphenolase activity were diminished by replacing the first activity controller with Thr. Crystals of this mutant (Ala243Thr), were used for *in crystallo* activity tests and in comparison to those of wild type *Md*PPO1 they exhibited slower activity rates with both dopamine and tyramine (Table 4).^[61]^

**Table 4:** Summary of the mutations to the first activity controller residue: effects on PPOs′ copper content and diphenolase/monophenolase activity. The modification of copper content and the activities′ rate are described compared to the wild type of the corresponding enzymes.

First activity controller (HisB_1_+1)					
Enzyme	Mutant	Diphenolase activity	Monophenolase activity	Copper %	Ref.
*Md*PPO1	Ala243Thr	slower (dopamine: *in crystallo* activity)	slower (tyramine: *in crystallo* activity)	n.i.	^[61]^
*To*PPO2	Gly240Thr	−4.0‐fold (4‐methylcatechol: 53.20 s^−1^) −14‐fold (dopamine: 6.85 s^−1^)	−42‐fold (*p*‐cresol: 0.56 s^−1^) −18‐fold (tyramine: 0.47 s^−1^)	n.i.	^[70]^
	Gly240Asn	−10‐fold (4‐methylcatechol: 19.66 s^−1^) −15‐fold (dopamine: 6.77 s^−1^)	−14‐fold (*p*‐cresol: 1.67 s^−1^) −9.0‐fold (tyramine: 0.92 s^−1^)	n.i.	
*To*PPO6	Thr250Gly	∼same (4‐methylcatechol and dopamine)	n.d. (*p*‐cresol and tyramine)	n.i.	
	Thr250Asn	−9.0‐fold (4‐methylcatechol: 6.78 s^−1^) −9.0‐fold (dopamine: 1.85 s^−1^)	n.d. (*p*‐cresol and tyramine)	n.i.	
*Jr*PPO1	Asn240Lys	−37‐fold (dopamine: 2.51 s^−1^) −103‐fold (l‐DOPA: 1.08 s^−1^)	−456‐fold (tyramine: 0.0542 s^−1^) n.d. (l‐tyrosine)	40	^[69]^
	Asn240Gly	+3.0‐fold (dopamine: 300 s^−1^)	−3.0‐fold (tyramine: 7.60 s^−1^)	50	^[75]^
*Jr*PPO2	Gly240Asn	−3.0‐fold (dopamine: 66.3 s^−1^)	+1.2‐fold (tyramine: 10.9 s^−1^)	60	
*Cg*AUS	Thr253Asp	∼same (dopamine: 530 s^−1^)	++ (tyramine: 2.14 s^−1^)	45	^[45]^
	Thr253Asn	+1.5‐fold (dopamine: 850 s^−1^)	++ (tyramine: 1.19 s^−1^)	84	
	Thr253Glu	+2.5‐fold (dopamine: 1394 s^−1^)	++ (tyramine: 0.21 s^−1^)	47	
	Thr253Gly	−0.5‐fold (dopamine: 337 s^−1^)	++ (tyramine: 0.07 s^−1^)	73	
	Thr253Ser	∼same (dopamine: 500 s^−1^)	++ (tyramine: 0.01 s^−1^)	53	
	Thr253Cys	−0.4‐fold (dopamine: 312 s^−1^)	++ (tyramine: 0.04 s^−1^)	57	
	Thr253Ala	−4.0‐fold (dopamine: 140 s^−1^)	++ (tyramine: 0.05 s^−1^)	56	
	Thr253Ile	−20‐fold (dopamine: 27 s^−1^)	n.d. (tyramine)	58	
	Thr253Lys	−28‐fold (dopamine: 20 s^−1^)	n.d. (tyramine)	5	
*Sg*TYR	Asn190Gln	−1900‐fold (l‐DOPA: 1.3 units/mg)	n.i.	45	^[57]^
*Vv*TYR	Gly241Asn	faster (4‐methylcatechol: SDS‐activity gel)	faster (*p*‐tyrosol and tyramine: SDS activity gel)	n.i.	^[72]^
*Bm*TYR	Asn205Ala	−8.0‐fold (l‐DOPA)	−9.0‐fold (l‐tyrosine)	70	^[74]^
	Asn205Asp	−8.0‐fold (l‐DOPA)	−9.0‐fold (l‐tyrosine)	60	

n.d.: no detected activity, n.i.: no information from the particular study, ++: generation of monophenolase activity that was not present in the wild type, entries in s^‐1^ provide the k_cat_ value, while units/mg refers to the specific activity.

In the TYR of *V. vinifera* (*Vv*PPO) the first activity controller Gly241 was mutated to Asn and the activity was examined with SDS‐PAGE activity gels. The monophenolase (tyrosol) and diphenolase (4‐methylcatechol) activity of Gly241Asn was higher than that of the wild type as the “activity bands” of the mutant developed faster and stronger on the gel than those of the wild type (Table 4).^[72]^ In TYR from *S. glaucescens* (*Sg*TYR) the first activity controller Asn190 was mutated to a Gln and the diphenolase activity of the mutant with l‐DOPA was impaired ∼1900‐fold (Table 4).^[57]^ In *Bm*TYR the first activity controller (Asn205) was converted to Ala and Asp (Asn205Ala and Asn205Asp). The monophenolase (l‐tyrosine) and diphenolase (l‐DOPA) activity of the two mutants decreased about ninefold for l‐tyrosine and about eightfold for l‐DOPA, whereas the copper content for Asn205Ala decreased to 70 % and for Asn205Asp to 60 % (Table 4).^[74]^

Prexler et al.^[70]^ tried to explain the differences between COs and TYRs by mutating the first activity controller (Gly240) of the *To*PPO2 (CO) to Thr and Asn, while that of *To*PPO6 (TYR, Thr250) was mutated to Gly and Asn. The monophenolase activity of *To*PPO2 mutant Gly240Thr with *p*‐cresol and tyramine reduced 42‐ and 18‐fold, respectively, while its diphenolase activity with 4‐methylcatechol and dopamine decreased four‐ and 14‐fold, respectively. The monophenolase activity of the *To*PPO2 mutant Gly240Asn, with *p*‐cresol and tyramine decreased approximately 14‐ and ninefold, respectively, while its diphenolase activity with 4‐methylcatechol and dopamine was reduced 10‐ and 15‐fold, respectively. The *To*PPO6 mutants Thr250Gly and Thr250Asn showed no activity with *p*‐cresol and tyramine. The diphenolase activity of the *To*PPO6 mutant Thr250Gly remained similar to that of the wild type, whereas the activity of Thr250Asn with 4‐methylcatechol and dopamine was reduced approximately ninefold compared to that of the wild type and Thr250Gly. Thus, the four mutations markedly affected the enzyme's activity; however, the mutants retained the same TYR/CO classification as the wild‐type enzymes.^[70]^

In *Jr*PPO1 the first activity controller Asn240 was mutated to Lys (Asn240Lys). The mutation dropped the enzyme's monophenolase activity on tyramine about 456‐fold and its diphenolase activity on dopamine and l‐DOPA was reduced 37‐fold and 103‐fold, respectively, compared with the corresponding activities of the wild‐type enzyme (Table 4).^[69]^ A recent mutagenesis study on *Jr*PPO1 (Asn240) and its isoenzyme *Jr*PPO2 (Gly240) attempted to transfer the activity of *Jr*PPO1 to *Jr*PPO2 and *vice versa* by exchanging the first activity controller residues.^[75]^ The *Jr*PPO1‐Asn240Gly mutant showed impaired monophenolase activity on tyramine, which was about three times lower than that of the wild type, while its diphenolase activity with dopamine was increased approximately threefold, compared with that of the wild type. In contrast, the *Jr*PPO2‐Gly240Asn mutant exhibited a slight increase in monophenolase activity with tyramine of 1.2‐fold, whereas its activity on the diphenolic substrate dopamine decreased about threefold (Table 4).^[75]^ A comprehensive mutagenesis study targeting the first activity controller (Thr253) of *Cg*AUS was carried out in order to convert the CO *Cg*AUS into a TYR. Nine mutants were produced and seven of them (Asp, Asn, Glu, Gly, Ser, Cys, and Ala) imparted the former CO with monophenolase activity (Table 4). The other two mutants (Ile and Lys) showed lower activities with dopamine compared to the wild type (The253) and responded only to diphenolic substrates. Among the mutants, those with residues containing a carboxylic acid or carboxamide in their side chains showed the highest activity rates with tyramine. The study suggested that the amino acid residues located at the HisB_1_+1 position interact with the neighboring HisB_1_ residue, affecting the rigidity/flexibility and basicity of this conserved residue (HisB_1_). The mutated residues Asp and Asn in Thr253Asp and Thr253Asn are ∼2.6 and ∼2.8 Å away from the HisB_1_ imidazole group, respectively. Thus, they assist HisB_1_ in finding the best position for activation (Asp) so that HisB_1_ can react as a base to deprotonate the incoming monophenol, thereby initiating the hydroxylation reaction (Figure 2).^[45]^ The designation first activity controller truly characterizes the position HisB_1_+1 in the type‐III copper enzymes. The majority of the mutants have an pronounced effect on the monophenolase and diphenolase activity (Table 4). The most significant finding is that mutations targeting the position HisB_1_+1, like the ones studied in *Cg*AUS, did, for the first time, generate monophenolase activity and consequently converted a CO to a TYR. Asp and Asn residues at the first activity controller position of *Cg*AUS influence the basicity of the adjacent conserved histidine (HisB_1_+1), which enhances the monophenolic substrate‘s deprotonation. Therefore, it becomes understandable why many TYRs (*Bm*TYR,^[18]^ *Jr*PPO1,^[52]^ *Ao*TYR,^[66]^ *Vv*PPO,^[59]^ *Ab*PPO3,^[76]^ *Hs*TYR^[26]^ and etc.) do carry an Asn at the HisB_1_+1 position. However, one should always keep in mind that parameters like substrate guiding and substrate specificity also affect the PPO activity. For example, in *Jr*PPO1, the Asn240Gly mutant did lose part of its monophenolase activity with tyramine, while the diphenolase activity with dopamine increased as the diphenolic substrate may more easily find a catalytically productive position due to the reduced steric interference from Gly as the first activity controller (Table 4).^[75]^

### 2.5 The second activity controller (HisB_2_+1)

#### 2.5.1 *Classical proposed roles of* the second activity controller

Among PPOs the amino acid residue at the position of the second activity controller (HisB_2_+1, Figure 3) varies more than that at the first activity controller (HisB_1_+1) position. Arg and Tyr are present at the HisB_2_+1 position in the COs *Ib*CO,[^37^, ^58^] *Cg*AUS,[^6^, ^11^] and *Ao*CO,^[67]^ whereas Leu, Ile, Arg, Asn, Phe, Gly, Val, Ser, and Asp are present at this position in the TYRs *Md*PPO1,^[15]^ *Sl*PPO1,^[63]^ *Jr*PPO1,^[52]^ *Bt*TYR,^[33]^ *Bm*TYR,^[18]^ *Ao*TYR,^[66]^ *Ab*PPO3,^[76]^ *Ab*PPO4,[^12^, ^22^] *Sc*TYR,^[47]^ *Manduca sexta* prophenoloxidase (*Ms*proPO),^[25]^ and *Marsupenaeus japonicas* prophenoloxidase (*Mj*proPO).^[64]^ Before the term second activity controller was coined this amino acid was described as a substrate‐guiding residue and substrate selector due to its influence on diphenolase/monophenolase activity.^[77]^

#### 2.5.2 Mutants of the second activity controller (HisB_2_+1)

The second activity controller was first examined in bacterial *Bm*TYR^[78]^ by mutating Arg209 to His (Arg209His). The conversion showed a negligible impact on both activities as the diphenolase (l‐DOPA) activity was reduced 1.5‐fold and the monophenolase (l‐tyrosine) activity was increased 1.7‐fold compared with the activities of wild‐type *Bm*TYR (Table 5).^[78]^ Prexler et al. characterized the second activity controller residue as a substrate selector in plant PPOs.^[77]^ In the eleven PPO isoenzymes from *T. officinale* (*To*PPOs), the second activity controller is either a positively charged Arg or a hydrophobic Ile. Reciprocal mutations of *To*PPO2 and *To*PPO6 were generated by exchanging the second activity controller between them. The positively charged Arg in wild‐type *To*PPO6_wt_ and *To*PPO2‐Ile244Arg mutant interacted with the negatively charged tail of the diphenolic substrate 3,4‐dihydroxyphenylacetic acid (DOPAC). On the other hand, wild‐type *To*PPO2_wt_ and the *To*PPO6‐Arg254Ile mutant, both of which carry an Ile at the second activity controller position, showed weaker interactions (increased *K*_M_ values) with DOPAC. In contrast to DOPAC, dopamine has a positively charged tail and thus led to opposite effects (lower *K*_M_ values) in the examined wild‐type and mutant *To*PPOs containing an Ile in the second activity controller (Table 5).^[77]^

**Table 5:** Summary of the mutations to the second activity controller residue: effects on PPOs′ copper content and diphenolase/monophenolase activity. The modification of copper content and the rate of activity are described compared to the wild type of the corresponding enzymes.

Second activity controller (HisB_2_+1)					
Enzyme	Mutant	Diphenolase activity	Monophenolase activity	Copper %	Ref.
*Bm*TYR	Arg209His	−1.5‐fold (l‐DOPA: 7.18 s^−1^) ∼same (d‐DOPA: 6.88 s^−1^)	+1.7‐fold (l‐tyrosine: 2.24 s^−1^)	n.i.	^[78]^
*To*PPO2	Ile244Arg	−1.77‐fold (4‐methylcatechol: 140 s^−1^) −4.14‐fold (catechol: 17.6 s^−1^) −3.12‐fold (dopamine: 66.5 s^−1^) −2.2‐fold (DOPAC: 85.2 s^−1^)	n.i.	n.i.	^[77]^
*To*PPO6	Arg254Ile	−1.61‐fold (4‐methylcatechol: 310 s^−1^) −1.87‐fold (catechol: 193 s^−1^) ∼same (dopamine: 89 s^−1^) −1.84‐fold (DOPAC: 40 s^−1^)	n.i.	n.i.	
*Jr*PPO1	Leu244Arg	−4.0‐fold (dopamine: 24.9 s^−1^) −11‐fold (l‐DOPA: 10.5 s^−1^)	−15‐fold (tyramine: 1.62 s^−1^)	50	^[69]^
*Cg*AUS	Arg257Gly	+2.3‐fold (dopamine: 1264 s^−1^)	n.d.	77	^[45]^
	Arg257Leu	+4.0‐fold (dopamine: 2245 s^−1^)	n.d.	65	
	Arg257Ile	+3.0‐fold (dopamine: 1660 s^−1^)	n.d.	66	
	Arg257Asp	+2.5‐fold (dopamine: 1380 s^−1^)	++ (tyramine: 8.26 s^−1^)	11	

n.d.: no detected activity, n.i.: no information from the particular study, ++: generation of monophenolase activity that was not present in the wild type, entries in s^‐1^ refer to k_cat_ values.

Moreover, the second activity controller (Leu244) of the TYR *Jr*PPO1 was mutated to an Arg, and the resulting mutant Leu244Arg was 15 times less active on the monophenol tyramine and about four and 11 times less active on the diphenols dopamine and l‐DOPA, respectively (Table 5).^[69]^

Very recently, four single mutants (Gly, Leu, Ile, and Asp) targeting the second activity controller (Arg257) of *Cg*AUS were prepared.^[45]^ Of the four mutants only Arg257Asp showed monophenolase activity with tyramine and l‐tyrosine, demonstrating that this mutation has converted the CO into a TYR. The diphenolase activity of the Arg257Asp mutant was increased approximately 2.5‐fold compared to that of the wild type. Notably, a similar effect was observed for the other three mutants, Arg257Gly, Arg257Leu, and Arg257Ile as also their diphenolase activity increased two‐, four‐, and threefold, respectively (Table 5).^[45]^ Similarly to the first activity controller, the second activity controller also influences both activities (Table 5). The Arg257Asp mutant of *Cg*AUS indicates that certain amino acids at this position can generate monophenolase activity and thereby convert a CO into a TYR. Asp at the second activity controller position influences the basicity of the adjacent conserved histidine (HisB_2_+1) in a manner similar to the first activity controller as described for the *Cg*AUS mutant (Arg257Asp).^[45]^ However, the substrate guiding effect of the second activity controller should not be disregarded. Prexler et al. showed that the PPO's affinity for the substrate is affected by the interactions between the second activity controller and the substrate's tail and therefore, especially with more effective inhibitors in mind, this position should also receive its due attention.

### 2.6 Thioether bridge

#### 2.6.1 *Classical proposed roles of* the thioether bridge

Most of the structurally known plant and fungal PPOs contain a thioether bond between the Cϵ atom of the second CuA coordinating histidine (HisA_2_) and the sulfur of a neighboring cysteine residue (Figure 3). This unusual bond does not occur in fungal *Ao*CO,^[67]^ bacterial and mammalian PPOs, and arthropod HCs, but is present in mollusk HCs.^[79]^ In most PPOs, the HisA_2_ of CuA is located on a flexible loop, whereas the remaining five His of the dicopper center are situated on α‐helices.^[52]^ The thioether bond therefore stabilizes the position of HisA_2_ and thereby structurally restricts the CuA position. This restriction optimizes the redox potential of the enzyme and allows for the fast electron transfer required for the catalytic reaction.^[58]^ Concerning monophenolase activity in plant PPOs, it has been suggested that the Phe gatekeeper residue and the thioether bridge prevent the substrate from performing a rotation that presumably is required for monophenol hydroxylation, whereas the oxidation of diphenols is still possible.[^18^, ^72^] As mentioned before (Section 2.2.1), this theory was refuted by the crystal structure of the plant TYRs *Jr*PPO1,[^51^, ^52^, ^60^] *Md*PPO1,[^15^, ^61^, ^62^] and *Sl*PPO1,^[63]^ which hydroxylate monophenols despite containing a Phe at the gatekeeper position and an intact thioether bridge. It was also suggested that the thioether bridge, together with the conserved disulfide bonds is involved in copper incorporation in plant and fungal PPOs.^[52]^ Biochemical and structural investigations on the holo‐ and apo‐form of *Sl*PPO1 showed that thioether bridge formation is related to the copper content and the exact position of the gatekeeper residue (Phe270).^[63]^ The holo‐form contains two copper ions in the active center, but lacks the conserved thioether bridge. In contrast, the apo‐form in which the copper ions have been chemically removed contains an intact thioether bridge. Depending on the absence or presence of the thioether bridge, the gatekeeper position significantly shifts between the apo‐ and holo‐structures. The absence of the thioether bridge in the holo‐structure allows the gatekeeper residue to exhibit high spatial flexibility, whereas the presence of the thioether bridge restrains this flexibility and thereby stabilizes the position of the gatekeeper residue.^[63]^ Based on docking results, a dual‐functionality for the gatekeeper residue of *Sl*PPO1 is proposed as Phe270 not only stabilized substrates by π‐stacking interactions but was also able to shield the active site from the approaching substrates.^[63]^

#### 2.6.2 Mutants of the thioether bridge constituent (Cys)

In *Ao*TYR (melO), Cys82 forms the thioether bond with the conserved His84 (HisA_2_). The mutant Cys82Ala lacking the thioether bond did lose monophenolase activity with l‐tyrosine and diphenolase activity with l‐DOPA (Table 6).^[56]^ In another study of a similar enzyme, *Ao*TYR (melB), the Cys92Ala mutant to the thioether bridge constituent Cys92, was able to form the oxy‐form after the addition of Cu^II^, while wild‐type *Ao*TYR could only form the met‐form after incubation with Cu^II^. In the Cys92Ala mutant the monophenolase activity with l‐tyrosine was reduced about 33‐fold.^[80]^ Both studies indicate that the thioether bond disruption impairs tyrosinase activity.

**Table 6:** Summary of the mutations to the thioether bridge constituent, the seventh His and the disulfide bonds: effects on PPOs′ copper content and diphenolase/monophenolase activity. The modification of copper content and the activities are described compared to the wild type of the corresponding enzymes.

Thioether bridge constituent					
Enzyme	Mutant	Diphenolase activity	Monophenolase activity	Copper %	Ref.
*Ao*TYR^[b]^	Cys92Ala	n.i.	−33‐fold (l‐tyrosine: 1.8 s^−1^)	n.i.	^[80]^
*Ao*TYR^[a]^	Cys82Ala	n.d. (l‐DOPA)	n.d. (l‐tyrosine)	60	^[56]^
*Cg*AUS	Cys97Ala	n.d. (butein and fisetin) −7845‐fold (4‐*tert*‐butylcatechol: 16 μmol/(l x min))	n.i.	100	^[53]^
	Cys97Ala	−95‐fold (dopamine: 5.84 s^−1^)	++ (tyramine: 0.14 s^−1^)	42	^[45]^
	Cys97Ser	−37‐fold (dopamine: 15 s^−1^)	++ (tyramine: 0.55 s^−1^)	59	
	Cys97Asp	−350‐fold (dopamine: 1.54 s^−1^)	++ (tyramine: 0.07 s^−1^)	53	
	Cys97Asn	−450‐fold (dopamine: 1.24 s^−1^)	++ (tyramine: 0.05 s^−1^)	46	
	Cys97Gly	−13‐fold (dopamine: 43 s^−1^)	++ (tyramine: 0.12 s^−1^)	34	
Seventh histidine (7th His)					
*Hs*TYR	His389Ala	n.d.	n.d.	0	^[54]^
	His389Ala	+1.1‐fold (l‐DOPA: 38.4 s^−1^)	∼same (l‐tyrosine: 0.63 s^−1^)	n.i.	^[55]^
*Cg*AUS	His285Ala	−38‐fold (dopamine: 14.7 s^−1^)	n.d. (tyramine)	23	^[45]^
*Ao*TYR^[a]^	His332Asn	n.d. (l‐DOPA)	n.d. (l‐tyrosine)	40	^[56]^
Disulfide bonds (S−S)					
*Cg*AUS	Cys31Ala	n.d. (dopamine)	n.d. (tyramine)	n.i.	^[45]^
	Cys32Ala	n.d. (dopamine)	n.d. (tyramine)	n.i.	
*Dm*proPO	Cys586Ser	−2.5‐fold (dopamine: 5752 s^−1^)	n.i.	n.i.	^[83]^
	Cys588Ser	−2.7‐fold (dopamine: 5333 s^−1^)	n.i.	n.i.	
	Cys586Ala‐Cys588Ala	−3.1‐fold (dopamine: 4595 s^−1^)	n.i.	n.i.	
	Cys586Ser‐Cys588Ser	−2.4‐fold (dopamine: 5958 s^−1^)	n.i.	n.i.	

[a] Refers to *Ao*TYR (mel0‐Q00234). [b] Refers to *Ao*TYR (melB‐Q2UP46). n.d.: no detected activity, n.i.: no information from the particular study, ++: generation of monophenolase activity that was not present in the wild type, entries with μmol/(l×min) refer to volumetric activity and those with s^‐1^ give the k_cat_ value.

In *Cg*AUS, Cys97 forms together with the conserved HisA_2_ (His116) the conserved thioether bond. Five mutants of *Cg*AUS targeting the thioether bridge Cys97Ala, Cys97Gly, Cys97Asn, Cys97Asp, and Cys97Ser exhibited monophenolase activity with tyramine. The activity rates varied depending on the substituting amino acid residue, with the Cys97Ser mutant exhibiting the highest rate (Table 6).^[45]^ In releasing HisA_2_ from the thioether bond, the mutant structurally resembles bacterial and mammalian PPOs with increased HisA_2_ flexibility and its resulting ability to move within the active site. In *Cg*AUS mutants the free HisA_2_ can approach the conserved waterkeeper residue (Glu) by ∼2.2 Å, which converts HisA_2_ to a base that deprotonates the incoming monophenolic substrates (Figure 3).^[45]^ Mutations of the thioether bridge exhibited different effects on monophenolase activity. In two fungal TYR (*Ao*TYR) studies[^56^, ^80^] the disruption of the thioether bridge reduced monophenolase activity. On the other hand, in the plant CO *Cg*AUS, breakage of the thioether bond produced monophenolase activity and converted *Cg*AUS to a TYR.^[45]^

### 2.7 The seventh His

#### 2.7.1 *Classical proposed roles of* the seventh His

The seventh His was first described in PPO from *Ralstonia solanacearum*, a gram‐negative, soil‐bound pathogenic bacterium.^[81]^ The seventh His is present in plant, fungal, and bacterial PPOs as well as in human TYR and is located before the third CuB‐coordinating histidine (HisB_3_‐1, Figure 3).[^26^, ^82^] Some other PPOs as well as the human tyrosinase‐related protein 1 and 2 (*Hs*TYRP1 and *Hs*TYRP2) have a Leu^[26]^ at this position, while insect PPOs festure a Val (*Ms*proPO^[25]^) or Trp (*Ag*proPO^[65]^). Notably, PPO enzymes harboring the seventh His at this position showed a high affinity for substrates with a carboxylic tail, whereas PPOs containing Leu at this position showed a higher affinity for substrates with a decarboxylated tail.[^81^, ^82^] The crystal structure of *Ab*PPO4 revealed the great flexibility of the CuB position[^49^, ^50^], wherefore the authors proposed that the seventh His restrains the apparently flexible CuB ion in *Ab*PPO4. In its alternative position in *Ab*PPO4, CuB is coordinated by the three conserved histidines and the seventh His, exhibiting a trigonal pyramidal (almost tetrahedral) coordination geometry.[^49^, ^50^]

#### 2.7.2 *Mutants of* the seventh His

In two different studies, the seventh His (His389) of *Hs*TYR was mutated to Ala. In the first study, the mutation led to the abolishment of tyrosinase activity and the loss of both copper ions from the active center (Table 6).^[54]^ In the second study, the mutation did not affect the enzyme's monophenolase activity with l‐tyrosine; however, the diphenolase activity with l‐DOPA was increased by 1.1‐fold compared to that of the wild type (Table 6).^[55]^ In *Cg*AUS, the seventh His (His285) was mutated to Ala, leading to a 38‐fold reduction in the diphenolase activity with dopamine (Table 6).^[45]^ The seventh His (His332) of *Ao*TYR (melO) was mutated to Asn and the enzyme lost its tyrosinase activity with the monophenolic substrate l‐tyrosine and the diphenolic substrate l‐DOPA (Table 6).^[56]^ In summary, substitutions of the seventh His in plant and fungal PPOs show a significant decrease in tyrosinase activity. Presumably the stability of the dicopper center of type‐III copper enzymes and in particular the position of the CuB ion is probably changed and therefore the hydroxylation and oxidation capability of the enzyme is diminished.

### 2.8 Disulfide bonds

#### 2.8.1 *Classical proposed roles for* the disulfide bonds

Plant PPOs contain two conserved disulfide bonds which keep the N‐terminal part of the active domain in place. Similarly, in insect PPOs, disulfide bonds connect the C‐terminal part to the active domain. In both cases, the disulfide bonds rigidify a flexible region of the active domain on the side distant from the shielding domain as the N‐terminal domain of insect PPOs has similar functions as the C‐terminal domain of plant PPOs.^[25]^ Mushroom PPOs usually contain a conserved motif CysXxxXxxCys (Xxx=any amino acid) whereby the two cysteines can form a conserved disulfide bond.^[50]^ PPOs and HCs that contain a shielding domain (N‐ or C‐terminal) presumably prevent direct access of a copper chaperone with copper ions to the active center. Thus, it is suggested that the motif CysXxxXxxCys at the C‐terminal domain represents a copper chaperone‐like machinery.^[50]^

Heterologously expressed *Md*PPO1 contains two of its three possible post‐translation modifications (i. e., two disulfide bonds and a thioether bridge), as demonstrated by mass spectrometry.^[15]^ The N‐terminal domain in the crystal structure of *Md*PPO1 is incomplete due to structural disorders caused by the lack of one of the two conserved disulfide bonds. Despite the missing disulfide bonds, the heterologously expressed *Md*PPO1 was still active.^[15]^ In contrast to recombinant *Md*PPO1, heterologously expressed *Sl*PPO1 and *Sl*PPO2 possess all three post‐translational modifications, as evidenced by mass spectrometry.^[63]^ However, as with *Md*PPO1, the N‐terminal domain in the crystal structures of apo‐ and holo‐*Sl*PPO1 are also incomplete due to structural disorders within the first 34 amino acids. This part is probably missing due to X‐ray radiation‐induced reduction of the bonds.^[63]^

#### 2.8.2 Mutants of the disulfide bond constituents (Cys‐Cys)

*Cg*AUS contains two conserved disulfide bonds, Cys31‐Cys94 and Cys12‐Cys32, and heterologous expression of the two mutants Cys31Ala and Cys32Ala failed in *Escherichia coli*, underlining the importance of these bonds for the correct folding of plant PPOs (Table 6).^[45]^

In a mutagenesis study on PPO1 from *Drosophila melanogaster* (*Dm*proPO), both disulfide bonds (Cys586–Cys630 and Cys588–Cys637) were disrupted by deleting one or both Cys residues of each bond. The two single mutations Cys586Ser and Cys588Ser, and the two double mutations Cys586Ala/Cys588Ala and Cys586Ser/Cys588Ser significantly reduced the enzyme's catalytic activity, as well as its thermostability and antibacterial activity, compared to those of the wild type (Table 6).^[83]^

### 2.9 Multiple mutation studies in PPOs

In *Ao*TYR (melO), five double mutants were designed targeting the six conserved histidines: His63Asn/His290Asn, His63Asn/His294Asn, His63Asn/His333Asn, His84Asn/His290Asn, and His93Asn/His290Asn. All mutants lost their monophenolase (l‐tyrosine) and diphenolase (l‐DOPA) activity (Table 7). In the same study, two more double mutants were prepared, one (Cys82Ala/His290Asn) targeting the thioether bridge‐forming Cys and the conserved HisB_1_ and another one (His63Ala/His332Ala) targeting the conserved HisA_1_ and the seventh His. Again, both double mutations eliminated tyrosinase activity (Table 7).^[56]^ The mutations of the conserved His prevent dicopper incorporation, which is demonstrated by the lack of copper ions and, therefore, eliminate the enzymes′ activity.

**Table 7:** Summary of the mutations to the thioether bridge constituent, the seventh His and the disulfide bonds: effects on PPOs′ copper content and diphenolase/monophenolase activity. The modification of copper content and the activities′ rate are described compared to the wild type of the corresponding enzymes.

Multi‐mutations					
Enzyme	Mutant	Diphenolase activity	Monophenolase activity	Copper %	Ref.
*Ao*TYR^[a]^	His63Asn‐His290Asn (HisA_1_‐HisB_1_)	n.d. (l‐DOPA)	n.d. (l‐tyrosine)	0	^[56]^
	His63Asn‐His294Asn (HisA_1_‐HisB_2_)	n.d. (l‐DOPA)	n.d. (l‐tyrosine)	0	
	His63Asn‐His332Asn (HisA_1_‐7thHis)	n.d. (l‐DOPA)	n.d. (l‐tyrosine)	0	
	His63Asn‐His333Asn (HisA_1_‐HisB_3_)	n.d. (l‐DOPA)	n.d. (l‐tyrosine)	0	
	Cys82Ala‐His290Asn (Th. bridge‐HisB_1_)	n.d. (l‐DOPA)	n.d. (l‐tyrosine)	0	
	His84Asn‐His290Asn (HisA_2_‐HisB_1_)	n.d. (l‐DOPA)	n.d. (l‐tyrosine)	0	
	His93Asn‐His290Asn (HisA_3_‐HisB_1_)	n.d. (l‐DOPA)	n.d. (l‐tyrosine)	0	
*Jr*PPO1	Asn240Lys‐Leu244Arg (HisB_1_+1‐HisB_2_+1)	−3200‐fold (dopamine: 0.029 s^−1^) −1070‐fold (l‐DOPA: 0.104 s^−1^)	n.d. (tyramine and l‐tyrosine)	55	^[69]^
	Asn240Thr‐Leu244Arg (HisB_1_+1‐HisB_2_+1)	−117‐fold (dopamine: 0.789 s^−1^) −618‐fold (l‐DOPA: 0.180 s^−1^)	n.d. (tyramine and l‐tyrosine)	45	
*Cg*AUS	Thr253Asp‐Arg257Asp (HisB_1_+1‐HisB_2_+1)	−3.0‐fold (dopamine: 171 s^−1^)	+generate (tyramine: 9.48 s^−1^)	44	^[45]^
	Thr253Asp‐Arg257Gly (HisB_1_+1‐HisB_2_+1)	+1.2‐fold (dopamine: 662 s^−1^)	+generate (tyramine: 1.91 s^−1^)	33	
	Thr253Gly‐Arg257Leu (HisB_1_+1‐HisB_2_+1)	∼same (dopamine: 535 s^−1^)	+generate (tyramine: 0.05 s^−1^)	34	
	Thr253Ser‐Arg257Gly (HisB_1_+1‐HisB_2_+1)	−1.3‐fold (dopamine: 430 s^−1^)	+generate (tyramine: 0.01 s^−1^)	33	
	Thr253Gly‐Arg257Val (HisB_1_+1‐HisB_2_+1)	−1.5‐fold (dopamine: 859 s^−1^)	+generate (tyramine: 0.02 s^−1^)	52	
	Thr253Gly‐Arg257Thr (HisB_1_+1‐HisB_2_+1)	−3.0‐fold (dopamine: 191 s^−1^)	+generate (tyramine: 0.56 s^−1^)	11	
	Thr253Asp‐Phe273Asp (HisB_1_+1‐gatekeeper)	−365‐fold (dopamine: 1.52 s^−1^)	+generate (tyramine: 0.01 s^−1^)	12	
	Thr253Asp‐Arg257Asp‐Phe273Asp (HisB_1_+1‐HisB_2_+1‐gatekeeper)	−200‐fold (dopamine: 2.76 s^−1^)	n.d. (tyramine)	18	
	Glu248Ala‐Thr253Glu (waterkeeper‐HisB_1_+1)	−567‐fold (dopamine: 0.98 s^−1^)	n.d. (tyramine)	1.2	
	Glu248Ala‐Phe273Glu (waterkeeper‐gatekeeper)	−471‐fold (dopamine: 1.18 s^−1^)	+generate (tyramine: 0.22 s^−1^)	1.7	
	Cys97Ala‐Thr253Asp‐Arg257Asp (thioether bridge‐HisB_1_+1‐HisB_2_+1)	−29‐fold (dopamine: 19 s^−1^)	+generate (tyramine: 6.52 s^−1^)	40	

[a] Refers to *Ao*TYR (mel0‐Q00234). n.d.: no detected activity, n.i.: no information from the particular study, ++: generation of monophenolase activity that was not present in the wild type, entries with s^‐1^ refer to the respective k_cat_ values.

On the other hand, double mutants targeting the first (Asn240) and the second (Leu244) activity controllers of *Jr*PPO1 (Asn240Lys/Leu244Arg and Asn240Thr/Leu244Arg) were not active anymore on the monophenols tyramine and l‐tyrosine, and the diphenolase activity of both double mutants was severely reduced, albeit the copper content remains similar to the wild type, indicating conversion of the TYR *Jr*PPO1 to a CO (Table 7).^[69]^

Moreover, six double mutations of *Cg*AUS targeting the two activity controllers Thr253 and Arg257 (Thr253Asp/Arg257Asp, Thr253Asp/Arg257Gly, Thr253Gly/Arg257Leu, Thr253Ser/Arg257Gly, Thr253Gly/Arg257Val, and Thr253Gly/Arg257Thr) were designed and imparted monophenolase activity with tyramine.^[45]^ Notably, the mutant in which both activity controllers were replaced with Asp (Thr253Asp/Arg257Asp) was most active on tyramine among the 39 mutants produced in this study (Table 7).^[45]^ The copper content of the six double mutants fluctuated from 10 to 50 % and showed that the two activity controllers influence the copper incorporation (Table 7). Moreover, formation of the oxy‐form with H_2_O_2_ was investigated, and the results asserted that all the six double mutants generate the oxy‐adduct. Similar to the single mutants (Sections 2.4 and 2.5) the double mutants corroborate the theory of control of tyrosinase activity by the two activity controllers in PPOs.

In the same study with *Cg*AUS, the monophenolase activity (tyramine) of the double mutant Thr253Asp/Phe273Asp, which targeted the gatekeeper residue Phe273 and the first activity controller Thr253, was 214 times lower than that of the single mutant Thr253Asp, while the triple mutant Thr253Asp/Arg257Asp/Phe273Asp, which aimed at the same residues, and in addition the second activity controller (Arg257), was completely inactive with tyramine (Table 7).^[45]^ Furthermore, the double mutant Glu248Ala/Thr253Glu of *Cg*AUS targeting the waterkeeper residue Glu248 and the first activity controller Thr253 showed a 567‐fold reduced diphenolase activity on dopamine compared to that of the wild type and no monophenolase activity. Another double mutant, Glu248Ala/Phe273Glu, targeting the waterkeeper Glu248 and the gatekeeper Phe273 residues showed clear monophenolase activity on tyramine and a 471‐fold reduced diphenolase activity on dopamine compared with that of the wild type (Table 7).^[45]^ The two double mutants have a vital effect on the activity of *Cg*AUS as both significantly impair the original diphenolase activity. The copper content of the two double mutants decreased significantly with final contents between 1 and 2 % (Table 7). The oxy‐form was not detected in any of the two mutants after using H_2_O_2_, probably due to the low copper incorporation. However, the *Cg*AUS double mutants Glu248Ala/Phe273Glu and Glu248Ala/Thr253Glu revealed that the waterkeeper‘s function is not necessarily linked to its exact conserved position in PPOs, especially strikingly demonstrated by the Glu248Ala/Phe273Glu mutant in which clear monophenolase activity was generated with tyramine. The *Cg*AUS triple mutant Cys97Ser/Thr253Asp/Arg257Asp which targets the thioether bond‐forming Cys97 and the two activity controllers Thr253 and Arg257 showed strong monophenolase activity with tyramine; however, the diphenolase activity was impaired by a factor of 29 compared to the wild type (Table 7)^[45]^ bringing those two activities quite close to each other with a diphenolase/monophenolase ratio of only 3. The copper content of the triple mutant was similar to the wild‐type and a weak peak at 345 nm indicated the formation of the oxy‐form.

## 3 Summary and Outlook

PPOs have been studied for more than a century, and 102 crystal structures have been published (as of August 2020). Despite a plethora of structural data on PPOs, it is still not possible to reliably predict the activity and thus the classification of a PPO based on its primary sequence or even its crystal structure. As crystallography has so far not been able to make a decisive contribution to answering the question “which residues affect the mode of action in type‐III copper enzymes?”, the focus now lies on comprehensive mutagenesis studies to determine the catalytic role of each relevant amino acid, including the six conserved histidines, the gatekeeper residue, the waterkeeper residue, the first (HisB_1_+1) and second (HisB_2_+1) activity controllers, the seventh His, the thioether bridge‐forming residues, and the conserved disulfide bonds. Mutagenesis studies on PPOs targeting these catalytically decisive residues have provided important insights into the catalytic mechanisms of TYRs and COs, and significantly improved our understanding of the subtle differences between TYR and CO activities. Three residues (the first and the second activity controllers, and the thioether bridge constituent) have a more crucial role, as they were shown to participate in the generation of monophenolase activity and thus are most likely to be responsible for the observed discrepancy between TYRs and COs. Specifically, amino acids like Asp and Asn at the position of the first and second activity controller residues can create an environment that allows the two conserved HisB_1_ and HisB_2_ residues to increase their basicity and react as proton acceptors. The clearly proven flexibility of the two copper ions enhances the mobility of HisB_1_ and HisB_2_, allowing them to move closer to the first and second activity controllers, respectively. The thereby activated HisB_1_ and HisB_2_ can deprotonate the candidate monophenolic substrates which may then react with the dicopper active center. On the other hand, the thioether bridge constituent binds the conserved HisA_2_ and keeps it stable next to CuA. However, HisA_2_ shares similar catalytic characteristics with HisB_1_ and HisB_2_ and, when released from the thioether bond, is also able to deprotonate phenolic substrates. The released HisA_2_ can move closer to the copper center and therefore to the conserved Glu waterkeeper. Interactions with this acidic residue let HisA_2_ become basic enough to deprotonate monophenolic substrates. Therefore, the conserved His have not only the structural role of copper coordination, but they also participate chemically in the hydroxylation of monophenolic substrates and it is consequently realistic to expect that PPOs with Asn or Asp as the first activity controller are TYRs.

For future work it is of utmost important that a standardized activity assays for PPOs is developed. In this review, we summarized and compared several PPO mutants that were characterized by different activity tests (kinetic measurements, specific activity, SDS‐PAGE activity gels, *in crystallo* activity tests, different substrates, different activity assays, etc.). We suggest that kinetic measurements are the most precise and accurate method for characterizing the activity of a PPO effectively and a standardized activity assays for PPOs should be followed by the PPO community. A good starting point for this endeavor would be the use of tyramine (monophenolic) and dopamine (diphenolic) as substrates as they are particularly soluble in water at high concentrations, and are readily accepted by the most PPOs. These two substrates can be used for the first kinetic measurements and, at the same time, serve as the basis for the classification of the examined enzyme (as either TYR or CO). 50 mM of HEPES or phosphate buffers at pH 7.0 may be used for the initial tests, while chelating buffers like citrate should be avoided. Pro‐ or active PPOs can be used, and SDS at a concentration in the range from 0.1 to 5 mM can activate the pro‐enzymes. The period of measurement depends on the reactivity of the enzyme; therefore, we suggest that before starting the actual measurements the reaction should be monitored for 24 hours to ascertain the acceptance of the substrate by the investigated enzyme and to find a suitable range of time for the determination of the pseudo‐zeroth order reaction rates. Then, optimal buffers, pH and SDS concentration should be determined and several additional substrates can be used to characterize the specificity of the investigated enzyme.

Additionally, it should be obligatory to determine the copper content (e. g., spectrophotometrically, by AAS, AES or ICP‐MS) as especially mutations close to the active center tend to influence the number of copper ions taken up by the catalytic cavity. We suggest the spectrophotometric method with 2,2’‐biquinoline as it can provide accurate results for the copper content and requires only a standard photometer.^[45]^ Moreover, the formation of the oxy‐form and the redox potential of the investigated enzymes will help to comprehensively characterize the type‐III dicopper center.

The production of active plant and mushroom PPOs remain an additional obstacle. The heterologous production of an active PPO at a level (mainly amount, enzymatic activity and purity) similar to pro‐PPO is currently not reported in the literature. However, it might be possible to construct mutated forms of pro‐PPOs and place a protease recognition sequence between the main and C‐terminal domains. The mutated pro‐enzyme can be expressed as described before, and subsequently, the two different domains may be separated by a specific proteolytic reaction.

Furthermore, PPOs have been successfully produced in the gram‐negative bacteria *E. coli* during the last decade. Using *E. coli* has been confirmed as one of the best options to produce high amounts of PPOs fast and at low costs.[^12^, ^15^] So far, the same PPO has not been tested for structural and biochemical equivalency of preparations produced in different expression systems (bacteria, yeast, insects, etc.) and this information is something that is missing from the bibliography.

Albeit the numerous mutagenesis studies included in this review, a saturation mutagenesis experiment targeting one of the discussed catalytic position has not been reported so far. The examination of all the amino acids in combination with molecular dynamics and the redox potential characterization will expand the biophysical and chemical characteristics of an investigated position and will elucidate the residue's impact on PPO activity.

Finally, to improve the understanding of structural key determinants of the monophenolase/diphenolase specificity, and to reduce the amount of experimental work, computational chemistry approaches should be considered. Molecular dynamics simulations could identify unbeknown motions or behavior of the herein discussed residues or even identify new decisive residues. Homology modeling might facilitate the search for specific structural features in novel PPOs. Thus, a combination of mutagenesis and electrochemistry should be applied to obtain valuable information on the redox potential of the dicopper site and to evaluate the effect of mutations on the environment of the metal center as local features, such as polarity, hydrophobicity, and electrostatics contribute to the chemistry of the active site. Thus, to fully decipher the monophenolase/diphenolase specificity of PPOs, an interdisciplinary cooperation between biochemists, computational chemists and electrochemists is required.

## Conflict of interest

The authors declare no conflict of interest.

## Biographical Information

*Ioannis Kampatsikas studied plant protection at the Aristotle University of Thessaloniki and completed his master's degree at BOKU Vienna, where he started his work on plant PPOs. In 2014, he joined Annette Rompel's research group at the University of Vienna to work toward his doctorate. Here he developed heterologous expression protocols for PPOs from several plants, paving the way for the identification of the amino acids responsible for C‐H activation in type‐III copper centers. He is currently examining peptides with proteolytic activities and analyzes this reaction‘s consequences for enzymes′ self‐activation processes*.

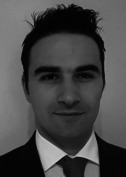

## Biographical Information

*Annette Rompel studied chemistry and received her doctorate at the Westfälische Wilhelms University of Münster. She was a visiting scientist at the University of California, Berkeley and the Lawrence Berkeley National Laboratory, RIKEN, the Institute of Physical and Chemical Research, Sendai, Japan, and the University of Southern Denmark in Odense. Since 2008, she is Head of the Department of Biophysical Chemistry at the University of Vienna. Her main research interests include the structure/function elucidation of type‐III copper proteins, the synthesis and characterization of polyoxometalates (POMs) with a focus on investigating POMs in emerging biological applications*.